# Pre-clinical study protocol: Blood transfusion in endotoxaemic shock

**DOI:** 10.1016/j.mex.2019.05.005

**Published:** 2019-05-09

**Authors:** Nchafatso G. Obonyo, Liam Byrne, John-Paul Tung, Gabriela Simonova, Sara D. Diab, Kimble R. Dunster, Margaret R. Passmore, Ai-Ching Boon, Louise See Hoe, Sanne Engkilde-Pedersen, Arlanna Esguerra-Lallen, Mohd H. Fauzi, Leticia P. Pimenta, Jonathan E. Millar, Jonathon P. Fanning, Frank Van Haren, Chris M. Anstey, Louise Cullen, Jacky Suen, Kiran Shekar, Kathryn Maitland, John F. Fraser

**Affiliations:** a Critical Care Research Group, The Prince Charles Hospital, Brisbane, QLD, Australia; b IDeAL/KEMRI-Wellcome Trust Research Programme, Kilifi, Kenya; c Australian Red Cross Blood Service, Kelvin Grove, Brisbane, Queensland, Australia; d The Canberra Hospital Intensive Care, Garran, ACT, Australia; e Australia National University, Canberra, ACT, Australia; f University of Queensland, Brisbane, QLD, Australia; g Queensland University of Technology, Brisbane City, QLD Australia; h School of Medical Sciences, Universiti Sains Malaysia Health Campus, Kelantan, Malaysia; i The University of Canberra, Bruce, ACT, Australia; j Sunshine Coast University Hospital Intensive Care, Birtinya, Qld, Australia; k Royal Brisbane and Women’s Hospital, Herston, QLD, Australia; l Adult Intensive Care, The Prince Charles Hospital, Chermside, Brisbane, QLD, Australia; m Department of Paediatrics, Faculty of Medicine, Imperial College London, United Kingdom

**Keywords:** Blood transfusion, Sepsis, Endotoxaemic shock, Packed red blood cells (PRBCs), Guidelines, Haemoglobin threshold, Storage duration

## Abstract

The Surviving Sepsis Campaign (SCC) and the American College of Critical Care Medicine (ACCM) guidelines recommend blood transfusion in sepsis when the haemoglobin concentration drops below 7.0 g/dL and 10.0 g/dL respectively, while the World Health Organisation (WHO) guideline recommends transfusion in septic shock ‘if intravenous (IV) fluids do not maintain adequate circulation’, as a supportive measure of last resort. Volume expansion using crystalloid and colloid fluid boluses for haemodynamic resuscitation in severe illness/sepsis, has been associated with adverse outcomes in recent literature. However, the volume expansion effect(s) following blood transfusion for haemodynamic circulatory support, in severe illness remain unclear with most previous studies having focused on evaluating effects of either different RBC storage durations (short versus long duration) or haemoglobin thresholds (low versus high threshold) pre-transfusion.

• We describe the protocol for a pre-clinical randomised controlled trial designed to examine haemodynamic effect(s) of early volume expansion using packed RBCs (PRBCs) transfusion (before any crystalloids or colloids) in a validated ovine-model of hyperdynamic endotoxaemic shock.
• Additional exploration of mechanisms underlying any physiological, haemodynamic, haematological, immunologic and tissue specific-effects of blood transfusion will be undertaken including comparison of effects of short (≤5 days) versus long (≥30 days) storage duration of PRBCs prior to transfusion.

## Graphical abstract

**Figure:**
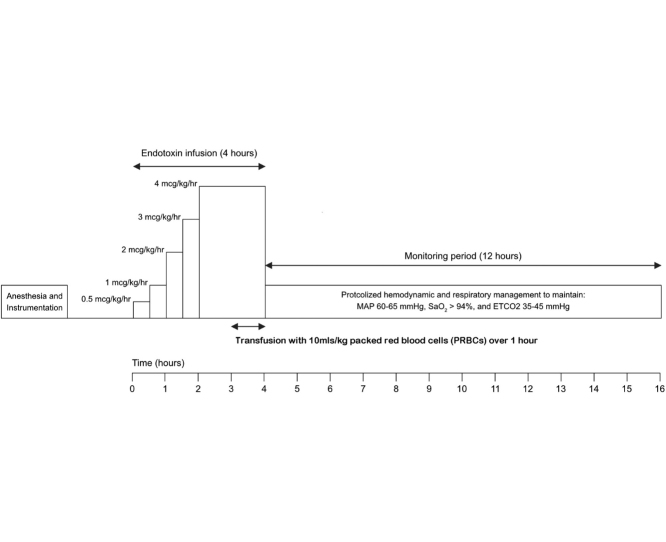

**Specifications Table**

**Table:** 

Subject Area:	Medicine and Dentistry
More specific subject area:	Critical Illness
Protocol name:	RESUS Transfusion Protocol
Reagents/tools:	Twenty six Merino ewes; Lipopolysaccharide (*E.coli* serotype O55:B5); Ovine blood for transfusion; Marquette Solar 8000 haemodynamic monitoring system; Oxford Optronix OxyFlow and OxyLite perfusion and oxygenation monitoring system; Powerlad 16/30 ADInstruments Data Acquisition System; ISCUS Flex Microdialysis Analyser; Philips ie.33 echo scanner and TomTec analysis software
Experimental design:	A randomised pre-clinical trial will be conducted in twenty-six, three-year old, non-pregnant Merino ewes. A two hit-hypothesis examining firstly the global effects of sepsis and secondly the attendant volume expansion effects resulting from blood transfusion will be explored. Sixteen sheep will undergo sepsis compared with non-septic controls (n = 10). Sheep will be divided into equal halves in the experimental (n = 8) and control groups (n = 5) with each sub-group receiving ovPRBC transfusion that is either fresh (≤5 days) or stored (≥30 days)
Trial registration:	PCTE0000156
Ethics:	Ethical approval has been sought and obtained from the Queensland University of Technology (QUT) Office of Research Ethics and Integrity (Certificate number 1400000032).

**Value of the Protocol**

**Table:** 

• Severe sepsis and septic shock are pro-haemolytic states that compromises the oxygen-carrying capacity of blood and is associated with high morbidity and mortality. • Blood transfusion and administration of blood-products are generally reserved as a late-stage treatment options in severe sepsis and septic shock after administration of fluids (crystalloids and colloids), vasopressor and inotropic agents. • Early administration of blood transfusion in severe sepsis and septic shock could improve oxygen delivery but the volume-expansion effects resulting from blood transfusion have not been studied.

## Description of protocol

### Background

Early recognition and source control, early blood cultures and commencement if antibiotic treatment as well as early haemodynamic resuscitation are key pillars of septic shock treatment.

### Introduction

The Surviving Sepsis Campaign (SSC) guidelines recommend initial haemodynamic resuscitation with crystalloid fluid (grade 1B evidence) [2,3] whereas use of colloids is recommended in patients who continue to require substantial amounts of crystalloids to maintain an adequate mean arterial pressure (MAP) (grade 2C evidence) [2,3]. Transfusion and administration of blood products in adults with sepsis is recommended when the haemoglobin concentration decreases to less than 7.0 g/dL with a target post-transfusion haemoglobin concentration of 7.0–9.0 g/dL (grade 1B evidence) [2,3]. These SSC guidelines further specify that this recommendation for administering blood products and transfusion is after resolution of tissue hypoperfusion, and also in the absence of extenuating circumstances, such as myocardial ischaemia, severe hypoxemia, acute haemorrhage, or ischaemic coronary artery disease [2,3]. The American College of Critical Care Medicine (ACCM), updated guideline recommends transfusion to a target haemoglobin of greater than 10.0 g/dL for septic shock among paediatric patients [4] and 7.0 g/dL target for stable critical illness without cardiopulmonary compromise [5]. The World Health Organisation (WHO) guideline recommends blood transfusion in septic shock as a supportive measure if intravenous (IV) fluids are insufficient to maintain adequate circulation [6]. Thus blood and blood-products are generally reserved as a later-stage treatment option in severe sepsis and septic shock after administration of fluids (crystalloids and colloids), vasopressor and inotropic agents.

Benefits of early goal-directed therapy (EGDT) including use of IV fluids for volume expansion and haemodynamic resuscitation in patients with severe sepsis and septic shock [7] could not be replicated in subsequent randomised controlled trials (RCTs) showing no mortality difference between EGDT and usual standard of care [[8], [9], [10], [11]]. These EGDT trials predominantly evaluated volume expansion using IV fluids and not blood transfusion. However, in the Protocolized Care for Early Septic Shock (ProCESS) multi-centre trial conducted across 31 hospitals in the United States of America, more patients in the EGDT-protocol group received packed red blood cell (PRBC) transfusion (14.4%) compared to the protocol-based standard care (8.3%) or usual care (7.5%) study groups (p = 0.001) but there were no significant differences in the overall outcomes evaluated across these treatment groups [8].

## Haemoglobin thresholds and transfusion

Majority of the studies on blood transfusion in severe illness including sepsis and septic shock have largely focused on evaluating the effects arising from differences in the RBC storage duration or haemoglobin levels pre-transfusion thus comparing either short- versus long-duration of RBC storage or low- versus high-haemoglobin thresholds. A multi-centre prospective cohort study examining PRBC transfusion in septic shock showed the haemoglobin level was the only measure that consistently differed between transfused and non-transfused patients and was also likely to be attributable to severity of the underlying disease or bleeding [12].

In 1999, the Transfusion Requirements In Critical Care (TRICC trial), reported a restrictive (Hb < 7.0 g/dL; n = 418) PRBC transfusion strategy is as effective as a liberal (Hb < 10.0 g/dL; n = 420) transfusion strategy in critical illness with no significant differences in 30-day mortality from all causes (p = 0.11) except for patients with active coronary ischaemic syndromes such as acute myocardial infarction and unstable angina [13]. The SSC guideline on maintaining haemoglobin levels of 7–9 g/dL in critical illness and only instituting RBC transfusion when the haemoglobin dropped below 7 g/dL [2,3], is based on recommendations from the TRICC trial [13]. In 2014, the Transfusion Requirements in Septic Shock (TRISS trial), showed similar outcomes with no 90-day mortality differences for lower (Hb < 7.0 g/dL) or higher (Hb < 9.0 g/dL) haemoglobin thresholds for transfusion in septic shock patients [14]. Surprisingly, this trial also revealed that significantly fewer transfusions (p < 0.001) were required in the lower-haemoglobin threshold group (n = 1,545) compared to the higher-haemoglobin threshold group (n = 3,088) [14]. In a 2015 systematic review with meta-analysis and sequential-analysis of randomized trials comparing restrictive versus liberal RBC transfusion strategies based on haemoglobin levels, Holst et al found restrictive transfusion strategies were associated with a reduction in both the number of RBC units and patients transfused concluding that liberal transfusion strategies did not convey any benefits to patients over restrictive strategies [15].

## RBC storage and transfusion

To minimize wastage of blood components, transfusion services typically provide the oldest compatible RBCs in a ‘*first in, first out*’ inventory management approach [16]. However, transfusion-associated morbidity and mortality may be exacerbated by RBC storage lesions, defined as biochemical and biomechanical changes in RBCs and the storage media used for ex-vivo preservation [17]. These changes in RBCs could render them ineffective oxygen carriers. *in vitro* studies have shown that many ‘early’ storage lesions found in donor blood stored for ≤14 days were reversible while those storage lesions associated with blood stored for ≥28 days were irreversible [18]. In practice, most PRBCs are stored for around 35–42 days with the longest PRBC shelf-life reported in clinical use being 49-days [19]. Recent large randomised controlled trials (RCTs) have not linked PRBC storage duration to adverse clinical outcomes. In 2015, the Age of Blood Evaluation (ABLE) RCT showed no decrease in the 90-day mortality among critically ill adults transfused with fresh (6.1 ± 4.9 days), versus standard-issue (22.0 ± 8.4 days) RBCs [16]. In 2017, Cooper et al reported the age of transfused RBCs did not affect 90-day mortality among a similar population of critically ill adults in the Transfusion versus Fresher Red-Cell Use in Intensive Care (TRANSFUSE) RCT that compared effects of short-term (11.8 ± 5.3 days) with long-term (22.4 ± 7.5) RBC storage [20]. Notably, febrile non-haemolytic transfusion reactions were reported to be more frequent following transfusion with short RBC storage in the TRANSFUSE trial, a finding which the authors contended needed further investigation to advance understanding on factors affecting quality of RBCs used for transfusion [20]. A previously published meta-analysis of 6 RCTs including 4,031 patients had reported no survival benefit associated with fresh PRBCs transfusion (≤10days) compared to standard practice of transfusing blood stored for 2–3 weeks [21]. A search on the International Standard Randomised Controlled Trials Number (ISRCT) online registry (http://www.isrctn.com/) for RCTs on blood transfusion in severe illness across low-and-middle income settings initially conducted on 13th March, 2018 (repeated on 30th October, 2018) found only one registered trial: Transfusion and treatment of severe anaemia in African children (TRACT, ISRCTN84086586), that commenced in September, 2014 [22].

Sepsis is a pro-haemolytic state that compromises the oxygen-carrying capacity of blood and the practice of administering blood transfusion and blood products after administration of crystalloid and colloid fluid boluses in septic shock could potentially further compromise oxygen delivery to tissues due to haemodilution. The overall haemodynamic and tissue-specific effects of volume expansion from blood transfusion have not been evaluated in light of the reported adverse effects of volume expansion with crystalloids and colloids in severe illness. We therefore propose to evaluate the physiological, haemodynamic, haematological, immunological, and tissue effects of blood storage and transfusion in septic shock using a validated pre-clinical ovine model of hyperdynamic endotoxaemic shock [1] and blood transfusion technique [23].

### Aims

The objectives of the study will be to examine the effects of early PRBC transfusion for volume expansion and haemodynamic resuscitation in endotoxaemic shock (instead of administering any crystalloids or colloids). Early administration of PRBC transfusion in ovine endotoxaemic shock will advance understanding on the potential effects of volume expansion from blood transfusion while eliminating the haemodilution arising from crystalloid and colloid fluid boluses that are usually administered prior to blood transfusion. This study will also explore mechanistic insights into differences seen in clinical studies with fresh versus stored blood transfusion comparing the global physiological, haematological, immunological and tissue-specific effects of fresh (≤5 days) versus stored (≥30 days) ovine PRBCs (ovPRBCs) administered in endotoxaemic shock.

This study will also involve detailed investigation and comparison of the biochemical properties of RBCs that have been stored for a short (≤5 days) versus a longer (≥30 days) duration prior to transfusion under controlled experimental conditions.

## Methods

### Study design and intervention groups

**Fig. 1:**
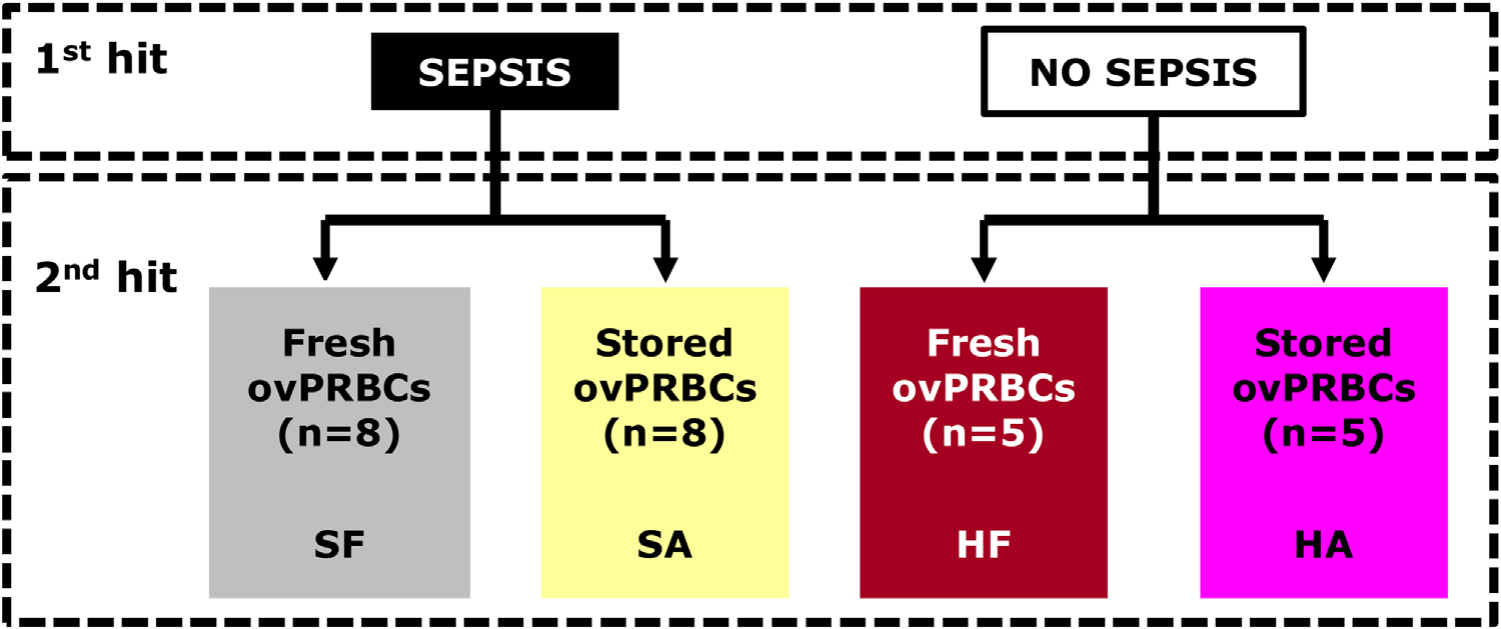
Pre-clinical trial study design examining a two-hit hypothesis of sepsis and transfusion with fresh versus stored ovine packed red blood cells (ovPRBCs). *Fresh ovPRBCs ≤5 days; stored ovPRBCs ≥30 days.****SF,****sepsis-fresh;****SA****, sepsis-aged;****HF****, healthy-fresh;****HA****, healthy-aged.*

A randomised pre-clinical trial will be conducted in twenty-six, three-year old, non-pregnant Merino ewes. A two hit-hypothesis examining firstly the global effects of sepsis and secondly the attendant volume expansion effects resulting from blood transfusion will be explored. Sixteen sheep will undergo sepsis compared with non-septic controls (n = 10). Sheep will be divided into equal halves in the experimental (n = 8) and control groups (n = 5) with each sub-group receiving ovPRBC transfusion that is either fresh (≤5 days) or stored (≥30 days) as shown in Fig. 1.

### Animal handling

A validated pre-clinical model of ovine hyperdynamic endotoxaemia has been developed by the Critical Care Research Group for this study [1].

In brief, sheep for the pre-clinical trial will be sourced from the Commonwealth Scientific and Industrial Research Organisation (CSIRO) and housed at the Queensland University of Technology- Medical Engineering Research Facility (QUT-MERF) with *ad libitum* access to food (proprietary sheep feed and Lucerne grass) and clean drinking water. Sheep will be handled by experienced, trained animal handlers at QUT-MERF in conformity with the Code of Practice for the Care and Use of Animals for Scientific Purposes published by the Australian Government’s National Health and Medical Research Council (NHMRC) [24].

### Anaesthetic induction

Prior to commencement of the experimental protocol, the animals will be fasted overnight for 12 h as routine practice prior to anaesthetic induction. All sheep will undergo anaesthesia, mechanical ventilation, surgical instrumentation as well as a one-hour period of resting and stabilisation post-instrumentation prior to commencement of the experimental procedure as previously described in the model development [1], Box 1.

Box 1 **Animal preparation (adapted from [1])**

At the start of the study, Seldinger technique will be used for percutaneous cannulation of the left external-jugular vein with a three lumen central-venous catheter (Arrow International, PA, USA) and a venous sheath (Edwards Lifesciences, CA, USA), both of which will be secured by a Vicryl stay-suture (Ethicon, GA, USA), under local anaesthesia.

Anaesthetic induction will be achieved using midazolam 0.5 mg/kg (Pfizer, WA, Australia), buprenorphine 300 mcg (Reckitt Benckiser Healthcare, Hull, United Kingdom) and alfaxalone 3 mg/kg (Jurox, NSW, Australia). Anaesthesia will be maintained throughout the experiment by infusion of alfaxalone 6 mg/kg/h (Jurox, NSW, Australia), midazolam 0.25 mg/kg/hr (Pfizer, WA, Australia), fentanyl 15 mcg/kg/h (Hameln Pharmaceuticals, Hameln, Germany) and ketamine 10 mg/kg/h (Troy Laboratories, NSW, Australia).

Endotracheal intubation with a cuffed endotracheal tube size 8–10 Fr (Smiths Medical International Hythe, Kent, UK) will be done to facilitate mechanical ventilation. Animals will be connected to a mechanical ventilator (Hamilton Medical AG, Switzerland) with mainstream end-tidal carbon dioxide (ETCO_2_) detection (Marquette TRAM, GE Healthcare, WI, USA). Ventilation tidal volume will be 10mls/kg and rate will be adjusted to maintain an ETCO_2_ of 35–45 mmHg. The fraction of inspired oxygen, FiO_2_, will be set to 30% and a positive end expiratory pressure, PEEP of 10 cmH_2_O at the start of the experiment. Thereafter, FiO_2_ and PEEP will be adjusted according to a pre-specified scale to maintain arterial oxygen saturations of ≥ 94% throughout the entire experiment.

Prior to surgical instrumentation all animals will be placed in the right-lateral position and remain so throughout the entire experiment. Each animal will receive one-litre of 5% dextrose (Baxter, IL, USA) administered at a rate of 250 mls/h to offset losses during overnight fasting and insensible losses during surgical instrumentation. Normothermia will be maintained throughout with a warming blanket (Hemotherm, Cincinnati Sub Zero, OH, USA). Gastric decompression will be achieved using a 14 Fr Salem Sump nasogastric tube (Coviden, MA, USA) left under free drainage. Continuous blood pressure monitoring and hourly blood sampling will be done via an arterial cannula (Vygon arterial leadercath, Ecouen, France) into a cut-down left facial artery. A pulmonary arterial catheter (Swan-Ganz CCOmbo, Edwards Lifesciences, CA, USA) will be inserted via the venous sheath (Edwards Lifesciences, CA, USA) and connected to a Vigilance II monitor (Edwards Lifesciences, CA, USA) for recording of continuous cardiac output (CCO), mixed venous oxygen saturation (SvO_2_), central venous pressure (CVP), pulmonary artery pressure (PAP), systemic vascular resistance (SVR) and core body temperature. A reference microdialysis catheter (CMA 64 MD probe Kista, Sweden) will be placed into a cut-down right femoral artery. Two myocardial microdialysis catheters (CMA 63 MD probe Kista, Sweden) will be inserted into the free wall of the left ventricle via a left-lateral thoracotomy in the 7^th^ intercostal space. One microdialysis catheter (CMA 63 MD probe Kista, Sweden) will be inserted into the left renal cortex and another similar microdialysis catheter into the right lobe of the liver via a left-lateral and midline laparotomy respectively. One cerebral microdialysis catheter (CMA 70 MD probe Kista, Sweden) will be inserted into the cerebral cortex via a 4mm left-hemisphere cranial burr-hole. All animals will receive Hartmann’s solution (Baxter, IL, USA) as maintenance fluid administered at a rate of 2.5 mls/kg/h for the duration of the entire experiment. All haemodynamic and ventilator data was automatically recorded with a data monitoring system (Solar 8000, GE Healthcare, WI, USA) and data captured every five seconds with customized software.

Alt-text: Box 1

### Experimental procedure

In the experimental group, sepsis will be induced by infusion of an escalating dose of lipopolysaccharide, LPS (*E.coli* serotype 055:B5) over 4-hs, according to validated and published criteria to mimic clinical hyperdynamic shock while avoiding severe acute pulmonary hypertension [1], Box 2.

Box 2 **Sepsis induction (Adapted from [1])**

Sepsis will be induced in the experimental groups of animals using an infusion of lipopolysaccharide, LPS (*E. coli* serotype O55:B5, diluted to 0.1 mcg/ml). In order to produce hyperdynamic shock, a dose-escalation of the LPS infusion will be administered starting at 0.5 mcg/kg/h for 30 min, then increasing to 1 mcg/kg/h for 30 min, 2 mcg/kg/h for 30 min, 3 mcg/kg/h for 30 min and then maintaining at 4 mcg/kg/h for a further 2 h (total LPS dose 11.25 mcg/kg).

Alt-text: Box 2

Three-hours from baseline, all animals will receive haemodynamic resuscitation with 10mls/kg packed red blood cell, ovPRBCs transfusion over one hour according to randomisation group (Fig. 1). Monitoring of the animals will be done for a period of 12-hs after completion of transfusion. Vasopressor support, if required, will be started under controlled administration using Gemini infusion (Alaris Medical Systems, CA, USA) and titrated to maintain a target mean arterial pressure, MAP of 60–65 mmHg. Noradrenaline (Hospira, IL, USA) will be the initial vasopressor of choice (60mcg/100 ml in 5% dextrose). If hypotension persists and the noradrenaline dose gets to 20mcg/min, then vasopressin (Pharmaceutical Partners of Canada Inc., ON, Canada) will be started at 0.8 units/hour and titrated upwards to a maximum dose of 1.6 units/hour, if required, to achieve the target MAP. Intra-venous sodium pentobarbitone (Virbac, NSW, Australia) will be administered at a dose of 162.5 mg/kg to euthanize all experimental and control animals at the end of the monitoring period. Autopsy will then be performed and tissue/organ samples will be retrieved and stored for further histological analysis.

### Ovine packed red blood cells (ovPRBCs) transfusion

Blood for transfusion will be obtained from a dedicated group of blood donors within the same flock of sheep at the Commonwealth Scientific and Industrial Research Organisation (CSIRO) facility. Blood collection and processing will be performed according to pre-specified validated criteria [23] by experienced, trained personnel collaborating on the study drawn from the Australian Red Cross Blood Service. Compatibility testing between donor ovPRBCs, and recipient sheep will be performed by saline and albumin agglutination methods [23]. Blood bags will be stored at 2–6 °C in the Critical Care Research Group pre-clinical facility and transferred to the QUT-MERF animal laboratory on the morning of the experiment. Investigators will be blinded to the duration of storage of the delivered blood bags. Prior to transfusion, a sample of blood will be drawn from the blood bag for full haemogram and biochemical testing. Transfusion with ovPRBCs will be done over one-hour at 10 mls/kg using standard intravenous giving-sets with 200 μm filter (Alaris CareFusion BD, Hampshire, UK).

### Sampling

Sample collection and organ retrieval in the experimental animals will be done as outlined in Table 1. Additionally, a sample of blood will be retrieved from each donor blood bags for testing prior to transfusion.

**Table 1:** Sampling schedule.

	Baseline (B)	Post-instrumentation (T1)	Post-sepsis (T2)	Post-transfusion (in hours)							
				0	0.5	1	2	3	6	9	12
BAL	√	√	√	√			√				√
WB	√	√	√	√	√	√	√	√	√	√	√
U	√	√	√	√	√	√	√	√	√	√	√
TS											√

**BAL**, bronchoalveolar lavage fluid; **WB**, whole blood; **U**, urine; **TS**, tissue samples.

### Outcome measures

Prior to transfusion, a sample will be retrieved from the donor ovPRBCs bag and evaluated on a Radiometer ABL800 Flex blood gas analyser (Radiometer, Copenhagen, Denmark) and a Coulter Act diff™ haematology analyser (Beckman Coulter Australia Pty Ltd, NSW, Australia) for quality control.

The primary outcome measure will be the physiological mean arterial blood pressure (MAP). This will be assessed by quantifying the vasopressor requirement to maintain the target MAP (60–65 mmHg) throughout the experiment. Secondary outcome measures include haematological parameters (full haemogram and differential count, arterial blood gas analysis, coagulation screen), haemodynamic parameters (pulmonary artery pressure, cardiac output, systemic vascular resistance, urine output, fluid balance), inflammatory cytokines, and tissue metabolism (lactate and pyruvate levels in the tissue microdialysate).

### Statistical analysis

All statistical analyses will be performed using STATA version 13 (StataCorp LLC, TX, USA) software package. The two-hit mechanism will be evaluated in stratified hypotheses testing the effects of sepsis versus control and the effects of fresh (≤5 days) versus stored (≥30 days) PRBCs transfusion. Ratios of lactate: pyruvate (L/P) in the microdialysate will be used to assess for impairment of oxidative metabolism in the different organs. All variables will be examined for normality and logartithmic transformation will be applied for non-normal distributions. Descriptive statistics for pooled variables will be presented by group and a paired *t*-test will be used to test the hypotheses and make comparison of the outcome measures across the groups (Fig. 1). Significance level for all statistical tests will be p < 0.05.

## Conclusion

Over the years, considerable advances have been made in pre-transfusion screening to mitigate risks associated with administration of blood and blood products. More recent research on blood transfusion has sought to examine whether clinical outcomes in hospitalised patients are affected by pre-transfusion haemoglobin level [13,14] or storage duration of transfused RBCs [16,20,25]. The recent finding of a higher frequency of febrile non-haemolytic transfusion following transfusion with short- compared to long-RBC storage duration in the TRANSFUSE trial [20] as well as extrapolation of the potentially harmful effects of volume expansion seen with fluids to blood transfusion warrant further mechanistic investigation.

Pre-clinical animal research is necessary to provide mechanistic understanding of disease pathophysiology as well as to evaluate safety and efficacy of treatment options. It also informs the design of future clinical trials aimed at improving patient outcomes. Blood transfusion in sepsis and septic shock is usually administered after a trial of crystalloid and colloid fluid boluses in addition to other therapies such as vasopressors, inotropes, antibiotics *et cetera*. Fluid boluses in severe illness have been associated with adverse outcomes in clinical practice [26] and could also cause haemodilution thus compromising the oxygen-carrying capacity of blood. This pre-clinical trial proposes to re-evaluate blood transfusion practices in septic shock to advance understanding towards improving clinical outcomes.
